# Identification of Cofragmented Combinatorial Peptide Isomers by Two-Dimensional Partial Covariance Mass Spectrometry

**DOI:** 10.1021/jasms.3c00111

**Published:** 2023-05-30

**Authors:** Taran Driver, Rüdiger Pipkorn, Vitali Averbukh, Leszek J. Frasinski, Jon P. Marangos, Marina Edelson-Averbukh

**Affiliations:** † Department of Physics, Imperial College London, London SW7 2AZ, U.K.; ‡ Department of Translational Immunology, German Cancer Research Centre, INF 580, 69120 Heidelberg, Germany

**Keywords:** mass spectrometry, 2D-PC-MS, combinatorial PTMs, histones, marker ion correlations

## Abstract

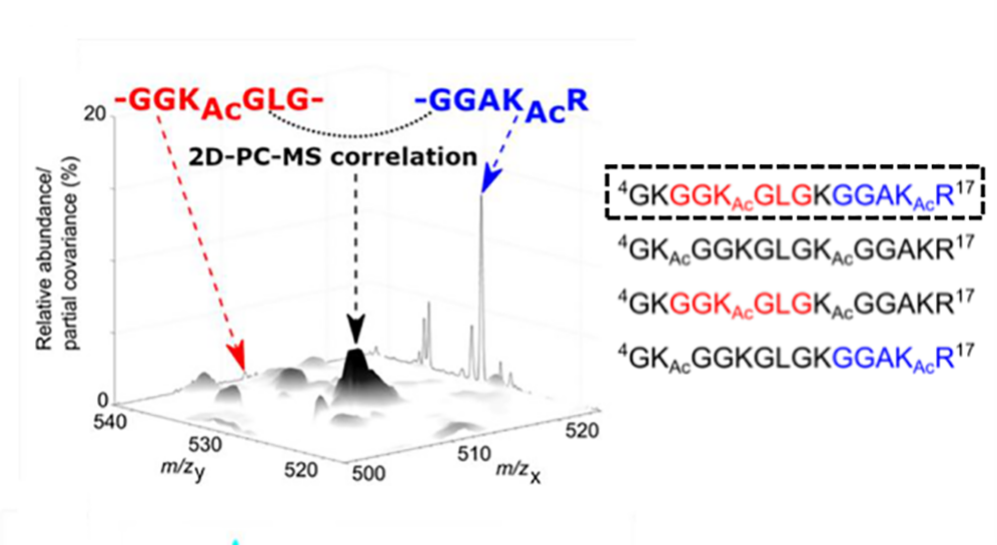

Combinatorial post-translational modifications (PTMs), such as those forming the so-called “histone code”, have been linked to cell differentiation, embryonic development, cellular reprogramming, aging, cancers, neurodegenerative disorders, *etc*. Nevertheless, a reliable mass spectral analysis of the combinatorial isomers represents a considerable challenge. The difficulty stems from the incompleteness of information that could be generated by the standard MS to differentiate cofragmented isomeric sequences in their naturally occurring mixtures based on the fragment mass-to-charge ratio and relative abundance information only. Here we show that fragment–fragment correlations revealed by two-dimensional partial covariance mass spectrometry (2D-PC-MS) allow one to solve the combinatorial PTM puzzles that cannot be tackled by the standard MS as a matter of principle. We introduce 2D-PC-MS marker ion correlation approach and demonstrate experimentally that it can provide the missing information enabling identification of cofragmentated combinatorially modified isomers. Our *in silico* study shows that the marker ion correlations can be used to unambiguously identify 5 times more cofragmented combinatorially acetylated tryptic peptides and 3 times more combinatorially modified Glu-C peptides of human histones than is possible using standard MS methods.

## Introduction

Combinatorial isomers are biomolecules which have the same chemical sequence and are modified by the same number of identical covalent modifications distributed differently across a series of possible modification sites. It has been shown that different combinatorial isomers regulate distinct cellular processes.^1^ One example is patterns of DNA methylation, which serve as important epigenetic actors in gene expression.^2^ The best-studied systems of combinatorial isomers are histone proteins, which can be heavily modified on their N-terminal tails.^3^ The proposition that modifications on the histone tails can act sequentially or in concert to code for different biological functions is known as the “histone code”.^4^

Mass spectrometry (MS) has proven to be a highly powerful method for the analysis of protein primary structure and the post-translational modifications.^5^ Nevertheless, identification of the commonly co-occurring combinatorial isomers of peptides and proteins presents a particular challenge to the state-of-the-art MS. Indeed, small differences in physical properties of the positional isomers frequently lead to their coelution and subsequent coisolation for tandem MS.^6^ As a result, as was shown by a rigorous mathematical analysis,^7^*m*/*z* degeneracy of the fragment ions produced by a series of cofragmented combinatorial isomers does not allow for a full set of unique “marker ions” characteristic of each individual isomer within an isomeric mixture, precluding identification of all the isomers.

There have been multiple reports dedicated to the MS-based histone code analysis and addressing the challenge posed by the cofragmented combinatorial isomers. For example, Phanstiel et al.^8^ solved systems of coupled linear equations to deconvolve the mixed spectra of coeluting histone structural isomers, but this method failed in more complex cases such as mixtures of variably diacetylated H4 histone tails (the third acetyl group was N-terminally fixed), solving which was conceded to be “mathematically impossible”.^8^ Feller et al.^9^ applied MS^3^ technique to identify cofragmented combinatorial isomers by marker ions arising from internal fragments. However, individual internal fragment marker ions do not always exist,^7^ including in the case of the diacetylated H4 histone peptides studied here. Abshiru et al.^10^ sought to overcome the standard MS information shortfall by exploiting the differences (frequently very subtle) in fragment signal intensities of individual combinatorial isomers. This approach, prohibitively requiring synthesis of a pure form of every individual isomer, also failed in the diacetylated H4 case. The alternative use of the spectral libraries^11^ leads to inherent ambiguity because of the variability and fluctuations of the experimental conditions. The more recently developed software packages, such as EpiProfile and EpiProfileLite,^12^ are based on the individual unique fragment ions and cannot overcome the *m*/*z* fragment degeneracy problem^7^ of many of the cofragmented positional isomers.

Here we demonstrate that using “marker ion *correlations*” (rather than “marker *ions*”), enabled by the two-dimensional partial covariance mass spectrometry (2D-PC-MS),^13^ allows one to resolve mixtures of isomeric combinatorially modified peptides which are not amenable to the existing MS approaches. The marker ion correlation method does not require any preliminary information on the MS/MS behavior of the individual isomers. The physical principle, measurement, and data processing procedures of 2D-PC-MS as well as the dedicated database search engine were reported previously.^13−15^ In the current work, we show that the fragment–fragment connectivity revealed by 2D-PC-MS can enable one to link pairs of otherwise non-isomer-specific individual fragments to a single possible isomeric sequence. We use the sequences of diacetylated positional isomers of histone H4 tryptic peptide 4–17 which presented a long-standing challenge to the state-of-the-art MS.^8,10^

## Materials and Methods

### Materials and Peptide Synthesis

Water, acetonitrile, and formic acid used for the MS analysis were of Optima LC–MS grade and were purchased from Fisher Scientific Ltd. Ammonium acetate was of LC–MS Chromasolv grade and manufactured by Fluka Analytical, and triethylamine was of analytical standard and purchased from Sigma-Aldrich Company Ltd.

For the solid-phase synthesis of all peptides, the 9-fluorenylmethoxycarbonyl (Fmoc) methodology^16^ was employed, using a fully automated multiple synthesizer (Syro II, Multi Syntech, Germany). The peptide synthesis was carried out on preloaded Wang resins. Peptide chain assembly was performed by the *in situ* activation of amino acid building blocks using 2-(1*H*-benzotriazol-1-yl)-1,1,3,3-tetramethyluronium hexafluorophosphate. The synthesized peptides were purified by preparative HPLC on a Kromasil (AkzoNobel/Sweden) 100-10C 10 μm 120 Å reversed-phase column (30 mm × 250 mm) using an eluent of 0.1% trifluoroacetic acid in water (A) and 80% acetonitrile in water (B). The peptides were eluted with a successive linear gradient of 25% B to 80% B in 30 min at a flow rate of 23 mL/min and lyophilized. The purified peptides were characterized with analytical HPLC and MS (Thermo Finnigan, LCQ).

### MS Analysis and Data Processing

2D-PC-MS measurements were performed using Thermo Fisher Scientific LTQ XL instrument. The instrument required no hardware modification. The MS/MS scans were performed at a scan rate of 125 000 Da/s, with AGC MSn target values of 100. The self-correcting partial covariance maps were built according to eq 3 of ref (13) using 10 000 microscans. For the 2D-PC-MS measurements, the synthetic peptides were dissolved in 50% acetonitrile/2% formic acid in water. The samples were infused into the mass spectrometer via a Harvard Apparatus 11 Plus single syringe pump coupled to a Nanospray II ion source (Thermo Fisher Scientific) at a flow rate of 3–5 μL/min and a spray voltage of 1.8–2.2 kV in positive ion mode and at a flow rate of 1 μL/min using no auxiliary desolvation gas. The temperature of the ion transfer capillary was held constant at 200 °C. The precursor ions of interest were fragmented by CID at normalized collision energies of 35%, with an activation time of 30 ms and a Mathieu q-value of 0.25.

The 2D-PC-MS data processing (see Figure 1 of ref (14)) was carried out using the in-house computer code written in Python (2.7) using numerical routines from the NumPy (http://www.numpy.org/) and SciPy (http://www.scipy.org/) libraries. The software, available at the GitHub repository https://github.com/TaranDriver/2D-PC-MS, reads in the MS/MS raw data in the text file format and calculates the TIC partial covariance (pCov) between each pair of *m*/*z* channels in the tandem mass spectra using eq 3 of ref (13). Another Python code was written for processing the resulting 2D-PC-MS maps to produce the scored lists of fragment ion correlations. This code first determined the features of a 2D-PC-MS map potentially corresponding to true correlation peaks, according to the height of their apexes, followed by the calculation of the 2D-PC-MS correlation score using eq C1 of ref (13). Marker ion correlations were identified manually.

### Marker Fragment *versus* Marker Ion Correlation Simulation

For the marker fragments *versus* marker ion correlation simulations shown in Figure 3, we constructed a Python code that considered all possible combinatorial isomers of the peptides. For each set of combinatorial isomers, we produced an exhaustive set of sequence-specific ions (for 1D MS/MS; terminal b and y ions) and fragment–fragment correlations (for 2D-PC-MS; terminal/terminal and terminal/internal correlations) which could be produced by the isomers. The code then counted the number of combinatorial isomers which produce either a 1D MS/MS or 2D-PC-MS signal which can uniquely identify a given isomer, *i.e.*, the peptide has no other combinatorial isomers which produce a signal with the same *m*/*z* value/pair of *m*/*z* values.

## Results and Discussion

**Figure 1:**
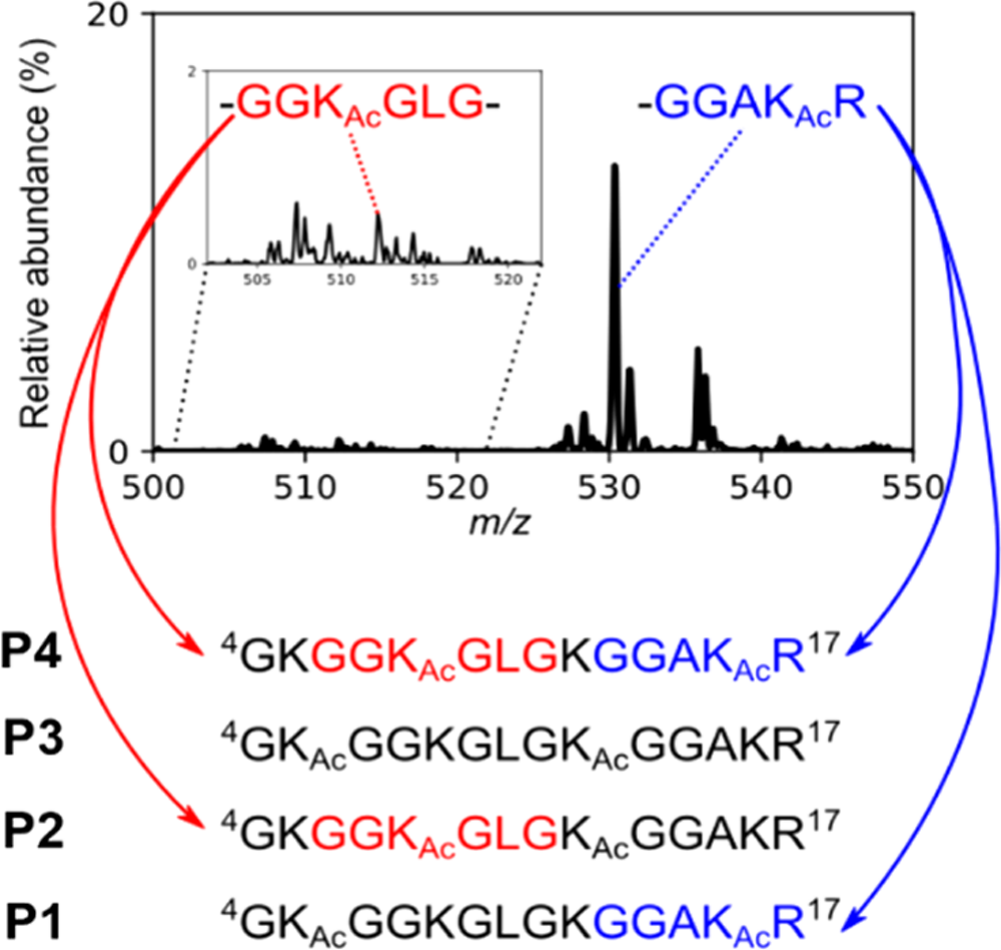
The *m*/*z* 500–550 region of the ion trap CID mass spectrum of a mixture of [M + 3H]^3+^ ions of combinatorial isomers P1–P4. Individual K_Ac_-containing fragments, such as GGK_Ac_GLG (b_i(3–8)_^+^, red) or GGAK_Ac_R (y_5_^+^, blue) can be produced by more than one combinatorial isomer, making it impossible, as a matter of principle, to identify all four cofragmented isomeric sequences.

The amino acid sequences of four diacetylated isomers of histone H4 tryptic peptide 4–17 carrying K_Ac_ residues at positions 5, 8, 12, and/or 16 (P1–P4)^16^ are presented in Figure 1. The sequences of the full series of diacetylated positional isomers (P1–P6) are given in Supporting Information (SI) section 1. Table S1 presents a theoretical analysis of all possible backbone fragment ions (terminal and internal) of P1–P6 that can be obtained during their dissociation, irrespective of the tandem MS method used. While the 5 and 8 and 12 and 16 diacetylated isomers of the ^4^GK_5_GGK_8_GLGK_12_GGAK_16_R^17^ peptide (P5 and P6 in Table S1) are straightforwardly identifiable under MS/MS based on their backbone marker ions^17^ (see Table S1), resolution of the isomeric mixtures containing P3 and/or P4 alongside the other isomers is not possible using the standard tandem MS. This is caused by the absence of any MS/MS fragment that would be unique to the location of the acetylated Lys in positions 5 and 12 and positions 8 and 16, respectively (see Table S1). Figure 1 shows a linear ion trap (LIT) CID spectrum of a mixture of the isomers P1–P4, where detection of the C-terminal fragment GGAK_Ac_R (y_5_^+^) provides evidence for the presence of either the K_5,Ac_K_16,Ac_ (P1) or K_8,Ac_K_16,Ac_ (P4) isomer without enabling one to differentiate between the two (see also Table S1). Equally, a detection of the internal fragment GGK_Ac_GLG (b_i(3–8)_^+^) can indicate the presence of either K_8,Ac_K_16,Ac_ (P4) or K_8,Ac_K_12,Ac_ (P2) but is unable to discriminate between the two isomers. In fact, in an arbitrary mixture of P4 with three other diacetylated forms P1–P3, the K_8,Ac_K_16,Ac_ isomer is unable to produce, regardless of fragmentation method (e.g., ETD or ECD, also within MS^*n*^ at *n* > 2) or instrumental mass accuracy, any possible unique fragment, either terminal or internal, that would distinguish it from the cofragmented isomers (see Tables S1 and S2), fully in line with the general mathematical treatment of ref (7) (see Theorem 2).

**Figure 2:**
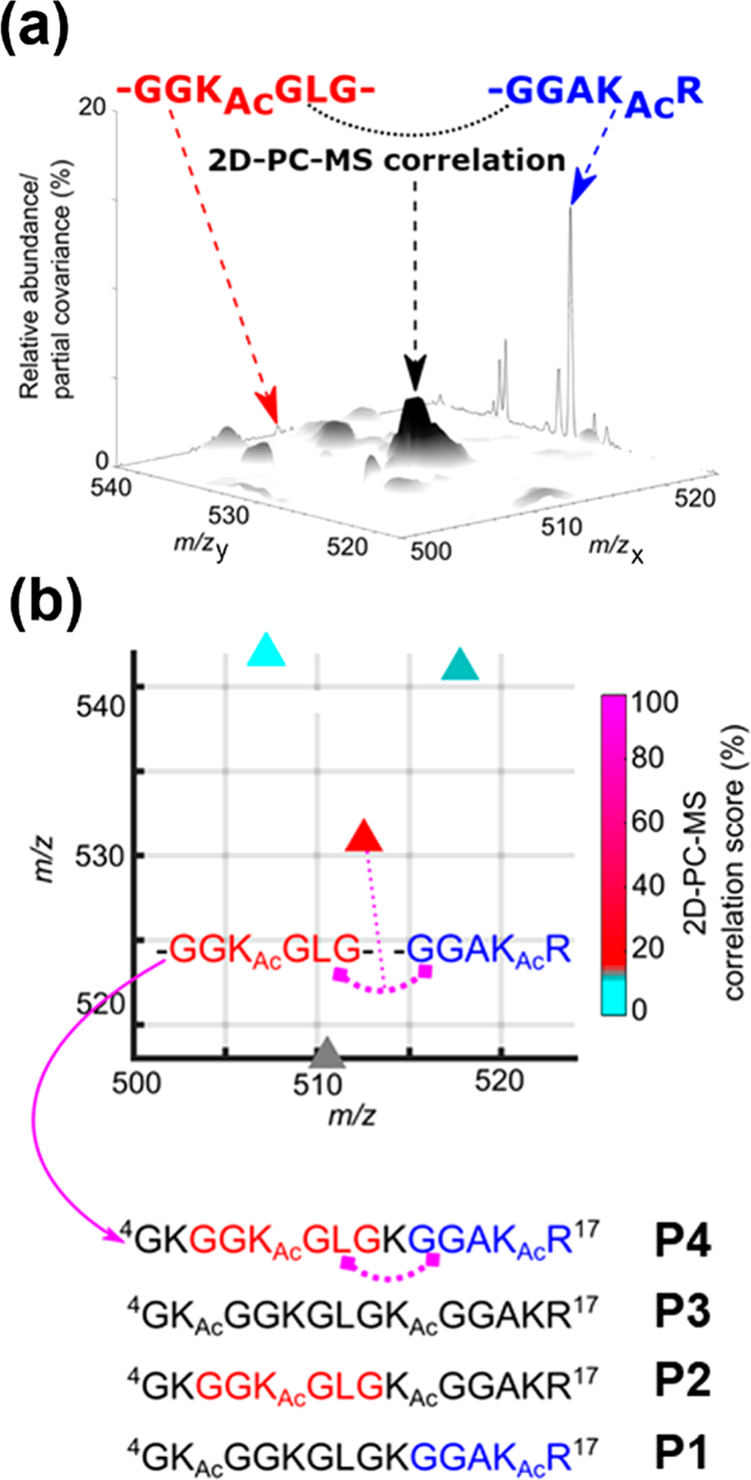
(a) The (*m*/*z* 500–524) × (*m*/*z* 518–542) region of 2D-PC-MS map measured for ion trap CID fragmentation of the [M + 3H]^3+^ ion mixture of P1–P4. The marker ion correlation signal of P4 between the *m*/*z* 512.2 GGK_Ac_GLG (b_i(3–8)_^+^, red) and *m*/*z* 530.3 GGAK_Ac_R (y_5_^+^, blue) fragments is shown. The back walls display the corresponding regions of the 1D CID spectrum shown in Figure 1. (b) Correlation scores^13^ of the main signals in the 2D-PC-MS map region shown in (a). The marker ion correlation peak of P4 is unambiguously distinguished based on its high correlation score.

In contrast to the standard MS/MS analysis, which inherently detects the GGK_Ac_GLG and GGAK_Ac_R fragments of cofragmeneted combinatorial isomers independently of each other, 2D-PC-MS enables one to establish their connection if they are produced from the same molecule. Figure 2 displays the correlation signal between the GGK_Ac_GLG and GGAK_Ac_R fragments on the 2D-PC-MS map (Figure 2a) measured for the mixture of four positional isomers P1–P4 under LIT-CID, unambiguously revealing the presence of the isomer P4 among the cofragmented isomers. Each signal of the 2D-PC-MS maps is scored by normalizing the peak volume to its standard deviation under jackknife resampling.^13^ The *m*/*z* 512.2 and *m*/*z* 530.3 correlation signal is identified as a true one by its high score, as displayed in Figure 2b. Thus, within 2D-PC-MS, the two *nonunique* individual fragments of P4 are linked to each other to produce the *unique* fragment–fragment correlation (marker ion correlation) of the particular combinatorial isomer. Further marker ion correlations measured for each of the four combinatorial isomers P1–P4 are shown in Figure S1.

Figure S2 illustrates the power of applying the 2D-PC-MS marker ion correlation approach to mixtures of two (P1, P2), three (P1–P3), and four (P1–P4) H4 peptide 4–17 isomers. For example, the y_5_^+^ and b_i(3–9)_^+^ correlation, being a marker of the isomer P3, is detected as a high-ranking signal only for the mixtures of three and four isomers (Figure S2b,c, respectively) but not for the mixture of two (Figure S2a), where P3 is absent. It was sufficient to interrogate one of the marker ion correlations per isomer to identify components of each of the analyzed mixtures. Moreover, since the volume of a fragment–fragment correlation is directly proportional to the concentration of the precursor ion (ref (13), Appendix A), the marker ion correlations can be used for quantifying the individual combinatorial isomers (see SI section 3).

**Figure 3:**
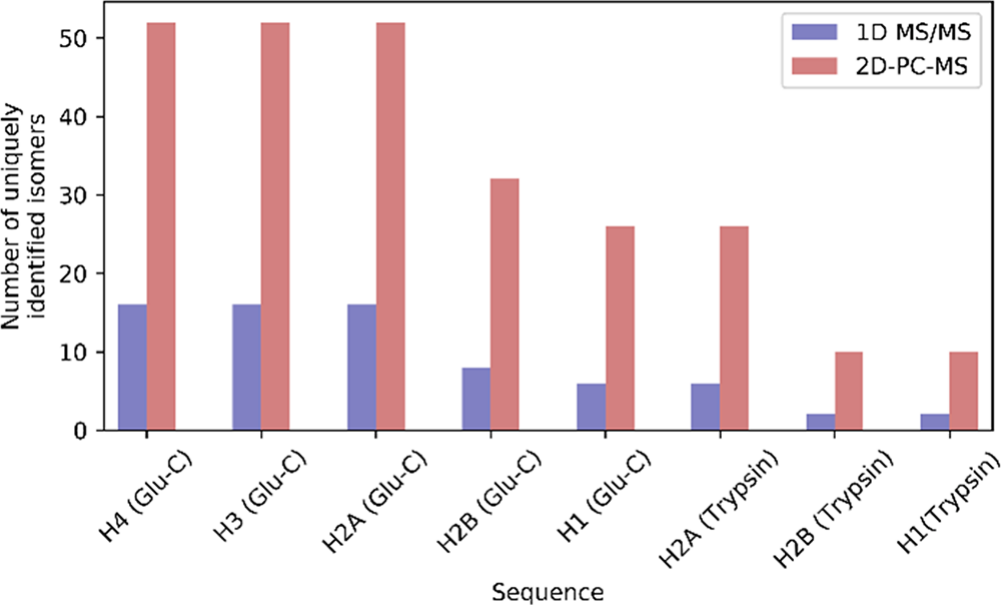
Total number of combinatorial isomers of acetylated Glu-C and tryptic peptides of human histones (derived from UniProtKB/Swiss-Prot database) that can be uniquely identified in their isomeric mixtures by the standard 1D MS/MS (blue) using the individual fragment ions *vs* 2D-PC-MS (red) using the marker ion correlations. The combinatorial isomer numbers that could be identified by 2D-PC-MS are 3 to 5 times higher than those that could be identified by 1D tandem MS.

We verified the generality of the marker ion correlation method *in silico* by identifying all possible 2D-PC-MS correlations unique to a given combinatorially modified peptide derived from UniProtKB/Swiss-Prot protein database human histone upon tryptic (bottom-up) or Glu-C (middle-down) digestion (see SI section 3 for details). The results shown in Figure 3 reveal that application of the 2D-PC-MS marker ion correlations can allow one to uniquely identify between 3 times (middle-down) and 5 times (bottom-up) more combinatorial isomers compared to what is theoretically possible via standard MS/MS. For isomers identified as one of two or three possible modified sequences the 2D-PC-MS enhancement factor reaches much higher values, see Figures S8 and S9.

## Conclusions

The new method of marker ion correlations within 2D-PC-MS enables one to resolve mixtures of cofragmented combinatorial isomers that could not be analyzed by the standard MS. This approach does not require *a priori* knowledge of the fragment mass spectra of any individual isomer^10,11^ and is therefore suitable for a discovery mode analysis. While demonstrated experimentally for mixtures of synthetic diacetylated positional isomers of histone H4 fragment 4–17, *in silico* simulations show that the marker ion correlation approach is general and can strongly enhance the number of cofragmented combinatorial isomer identifications relative to what is possible theoretically within the standard MS. Moreover, the marker ion correlation method can be used for quantifying the combinatorial isomers and can be straightforwardly extended to the analysis of positional isomers of other biopolymers, such as methylated oligonucleotides.

## Supporting Information Available

Analysis of uniqueness of theoretical fragments of positional isomers of diacetylated ^4^GK_5_GGK_8_GLGK_12_GGAK_16_R^17^ (Tables S1 and S2); 2D-PC-MS experimental data for mixtures of cofragmented diacetylated isomers (Figures S1 and S2); quantitative analysis of the combinatorial isomer mixture (Figure S3); *in silico* identification of MS/MS marker fragments and 2D-PC-MS marker fragment–fragment correlations (Table S3 and Figures S4–S9) (PDF)
