# Supplementary material for: Identification of Cofragmented Combinatorial Peptide Isomers by Two-Dimensional Partial Covariance Mass Spectrometry
Source: J Am Soc Mass Spectrom. 2023 May 30;34(7):1230–4. doi: 10.1021/jasms.3c00111 (PMC10326914; doi:10.1021/jasms.3c00111)
Supplement: Supplementary file 1 — js3c00111_si_001.pdf [file js3c00111_si_001.pdf]

Identification of Co-Fragmented Combinatorial Peptide Isomers by Two-Dimensional Partial Covariance Mass Spectrometry

Taran Driver[†], Rüdiger Pipkorn[‡], Vitali Averbukh[†], Leszek J. Frasinski[†], Jon P. Marangos[†], Marina Edelson-Averbukh[†,*]

[†]*Department of Physics, Imperial College London, SW7 2AZ London, UK*
[‡]*German Cancer Research Centre, Department of Translational Immunology, INF 580, 69120 Heidelberg, Germany*

Supporting Information

1. Analysis of uniqueness of theoretical b, c, a, y, z and x fragments (terminal and internal) that could be produced under MS/MS from positional isomers of diacetylated histone H4 fragment 4–17, $^{4}GK_{5}GGK_{8}GLGK_{12}GGAK_{16}R^{17}$

Table S1. All theoretically possible fragment ions that can be produced by backbone cleavages of the six positional isomers P1–P6. Rows contain all theoretically possible fragments (independent of fragmentation technique used, e.g. CID or ETD/ECD etc) that could be generated by one or multiple backbone cleavages of a given positional isomer. The symbols ‘x’ (blue cells) or ‘o’ (pink cells) across a row denote non-unique (identical or isomeric) fragments that can be produced from two or more positional isomers. The table demonstrates the absence of *any* unique 1D fragment, for the positional isomers **P3** and **P4** under *any* theoretical backbone fragmentation. A tick (yellow cells) denotes a unique 1D signal channel for **P1**, **P2**, **P5** and **P6**, however for **P1** and **P2** these amount to a selected few internal ions of which no 1D signal that could potentially correspond to such a fragment appears above ~2% RA in our standard 1D CID measurement of the **P1**–**P4** mixture. The *m/z* values of the listed fragments of **P1-P4**, which in contrast to P5 and P6, do not possess any unique terminal fragments are presented in Table S2.

Fragment ion	**P1** $K5_{Ac}K16_{Ac}$	**P2** $K8_{Ac}K12_{Ac}$	**P3** $K5_{Ac}K12_{Ac}$	**P4** $K8_{Ac}K16_{Ac}$	**P5** $K5_{Ac}K8_{Ac}$	**P6** $K12_{Ac}K16_{Ac}$
$[b/c/a]_{2}$	x	o	x	o	x	o
$[b/c/a]_{3}$	x	o	x	o	x	o
$[b/c/a]_{4}$	x	o	x	o	x	o
$[b/c/a]_{5}$	x	x	x	x	✓	✓
$[b/c/a]_{6}$	x	x	x	x	✓	✓
$[b/c/a]_{7}$	x	x	x	x	✓	✓
$[b/c/a]_{8}$	x	x	x	x	✓	✓
$[b/c/a]_{9}$	o	x	x	o	x	o
$[b/c/a]_{10}$	o	x	x	o	x	o
$[b/c/a]_{11}$	o	x	x	o	x	o
$[b/c/a]_{12}$	o	x	x	o	x	o
$[b/c/a]_{13}$	x	x	x	x	x	x
$[y/z/x]_{1}$	x	x	x	x	x	x
$[y/z/x]_{2}$	o	x	x	o	x	o
$[y/z/x]_{3}$	o	x	x	o	x	o
$[y/z/x]_{4}$	o	x	x	o	x	o
$[y/z/x]_{5}$	o	x	x	o	x	o
$[y/z/x]_{6}$	x	x	x	x	✓	✓
$[y/z/x]_{7}$	x	x	x	x	✓	✓
$[y/z/x]_{8}$	x	x	x	x	✓	✓
$[y/z/x]_{9}$	x	x	x	x	✓	✓
$[y/z/x]_{10}$	x	o	x	o	x	o
$[y/z/x]_{11}$	x	o	x	o	x	o
$[y/z/x]_{12}$	x	o	x	o	x	o
$[y/z/x]_{13}$	x	x	x	x	x	x
$[b/c/a]_{i(2-3)}$	x	o	x	o	x	o
$[b/c/a]_{i(2-4)}$	x	o	x	o	x	o
$[b/c/a]_{i(2-5)}$	x	x	x	x	✓	✓
$[b/c/a]_{i(2-6)}$	x	x	x	x	✓	✓
$[b/c/a]_{i(2-7)}$	x	x	x	x	✓	✓
$[b/c/a]_{i(2-8)}$	x	x	x	x	✓	✓

[*]m.edelson-averbukh@imperial.ac.uk

[b/c/a]$_{i(2-9)}$	o	x	x	o	x	o
[b/c/a]$_{i(2-10)}$	o	x	x	o	x	o
[b/c/a]$_{i(2-11)}$	o	x	x	o	x	o
[b/c/a]$_{i(2-12)}$	o	x	x	o	x	o
[b/c/a]$_{i(2-13)}$	x	x	x	x	x	x
[b/c/a]$_{i(3-4)}$	x	x	x	x	x	x
[b/c/a]$_{i(3-5)}$	x	o	x	o	o	x
[b/c/a]$_{i(3-6)}$	x	o	x	o	o	x
[b/c/a]$_{i(3-7)}$	x	o	x	o	o	x
[b/c/a]$_{i(3-8)}$	x	o	x	o	o	x
[b/c/a]$_{i(3-9)}$	✓	✓	x	x	x	x
[b/c/a]$_{i(3-10)}$	✓	✓	x	x	x	x
[b/c/a]$_{i(3-11)}$	✓	✓	x	x	x	x
[b/c/a]$_{i(3-12)}$	✓	✓	x	x	x	x
[b/c/a]$_{i(3-13)}$	x	o	x	o	x	o
[b/c/a]$_{i(4-5)}$	x	o	x	o	o	x
[b/c/a]$_{i(4-6)}$	x	o	x	o	o	x
[b/c/a]$_{i(4-7)}$	x	o	x	o	o	x
[b/c/a]$_{i(4-8)}$	x	o	x	o	o	x
[b/c/a]$_{i(4-9)}$	✓	✓	x	x	x	x
[b/c/a]$_{i(4-10)}$	✓	✓	x	x	x	x
[b/c/a]$_{i(4-11)}$	✓	✓	x	x	x	x
[b/c/a]$_{i(4-12)}$	✓	✓	x	x	x	x
[b/c/a]$_{i(4-13)}$	x	o	x	o	x	o
[b/c/a]$_{i(5-6)}$	x	o	x	o	x	o
[b/c/a]$_{i(5-7)}$	x	o	x	o	x	o
[b/c/a]$_{i(5-8)}$	x	o	x	o	x	o
[b/c/a]$_{i(5-9)}$	✓	✓	x	x	x	x
[b/c/a]$_{i(5-10)}$	✓	✓	x	x	x	x
[b/c/a]$_{i(5-11)}$	✓	✓	x	x	x	x
[b/c/a]$_{i(5-12)}$	✓	✓	x	x	x	x
[b/c/a]$_{i(5-13)}$	x	o	x	o	x	o
[b/c/a]$_{i(6-7)}$	x	x	x	x	x	x
[b/c/a]$_{i(6-8)}$	x	x	x	x	x	x
[b/c/a]$_{i(6-9)}$	o	x	x	o	o	x
[b/c/a]$_{i(6-10)}$	o	x	x	o	o	x
[b/c/a]$_{i(6-11)}$	o	x	x	o	o	x
[b/c/a]$_{i(6-12)}$	o	x	x	o	o	x
[b/c/a]$_{i(6-13)}$	x	x	x	x	✓	✓
[b/c/a]$_{i(7-8)}$	x	x	x	x	x	x
[b/c/a]$_{i(7-9)}$	o	x	x	o	o	x
[b/c/a]$_{i(7-10)}$	o	x	x	o	o	x
[b/c/a]$_{i(7-11)}$	o	x	x	o	o	x
[b/c/a]$_{i(7-12)}$	o	x	x	o	o	x
[b/c/a]$_{i(7-13)}$	x	x	x	x	✓	✓
[b/c/a]$_{i(8-9)}$	o	x	x	o	o	x
[b/c/a]$_{i(8-10)}$	o	x	x	o	o	x
[b/c/a]$_{i(8-11)}$	o	x	x	o	o	x
[b/c/a]$_{i(8-12)}$	o	x	x	o	o	x
[b/c/a]$_{i(8-13)}$	x	x	x	x	✓	✓
[b/c/a]$_{i(9-10)}$	o	x	x	o	o	x
[b/c/a]$_{i(9-11)}$	o	x	x	o	o	x
[b/c/a]$_{i(9-12)}$	o	x	x	o	o	x
[b/c/a]$_{i(9-13)}$	x	x	x	x	✓	✓
[b/c/a]$_{i(10-11)}$	x	x	x	x	x	x
[b/c/a]$_{i(10-12)}$	x	x	x	x	x	x
[b/c/a]$_{i(10-13)}$	o	x	x	o	o	x
[b/c/a]$_{i(11-12)}$	x	x	x	x	x	x
[b/c/a]$_{i(11-13)}$	o	x	x	o	o	x
[b/c/a]$_{i(12-13)}$	o	x	x	o	o	x

Table S2. Mass-to-charge ratios of all theoretically possible fragments that can be produced by all possible backbone cleavages of the four positional isomers P1–P4. Rows denote the *m/z* values of all theoretically possible fragments that can be generated (independently of fragmentation technique used, e.g. CID or ETD/ECD, also within MS^n, n>2) following one or multiple backbone cleavages of the individual positional isomers (denoted by columns).

Fragment ion	**P1**<br>K5$_{Ac}$K16$_{Ac}$	**P2**<br>K8$_{Ac}$K12$_{Ac}$	**P3**<br>K5$_{Ac}$K12$_{Ac}$	**P4**<br>K8$_{Ac}$K16$_{Ac}$
[b/c/a]$_{2}$	228.1343 / 243.1452 / 200.1394	186.1237 / 201.1346 / 158.1288	228.1343 / 243.1452 / 200.1394	186.1237 / 201.1346 / 158.1288
[b/c/a]$_{3}$	285.1558 / 300.1666 / 257.1608	243.1452 / 258.156 / 215.1502	285.1558 / 300.1666 / 257.1608	243.1452 / 258.156 / 215.1502
[b/c/a]$_{4}$	342.1772 / 357.1881 / 314.1823	300.1666 / 315.1775 / 272.1717	342.1772 / 357.1881 / 314.1823	300.1666 / 315.1775 / 272.1717
[b/c/a]$_{5}$	470.2722 / 485.2831 / 442.2773	470.2722 / 485.2831 / 442.2773	470.2722 / 485.2831 / 442.2773	470.2722 / 485.2831 / 442.2773
[b/c/a]$_{6}$	527.2936 / 542.3045 / 499.2987	527.2936 / 542.3045 / 499.2987	527.2936 / 542.3045 / 499.2987	527.2936 / 542.3045 / 499.2987
[b/c/a]$_{7}$	640.3777 / 655.3886 / 612.3828	640.3777 / 655.3886 / 612.3828	640.3777 / 655.3886 / 612.3828	640.3777 / 655.3886 / 612.3828
[b/c/a]$_{8}$	697.3992 / 712.4101 / 669.4042	697.3992 / 712.4101 / 669.4042	697.3992 / 712.4101 / 669.4042	697.3992 / 712.4101 / 669.4042
[b/c/a]$_{9}$	825.4941 / 840.505 / 797.4992	867.5047 / 882.5156 / 839.5098	867.5047 / 882.5156 / 839.5098	825.4941 / 840.505 / 797.4992
[b/c/a]$_{10}$	882.5156 / 897.5265 / 854.5207	924.5262 / 939.5371 / 896.5313	924.5262 / 939.5371 / 896.5313	882.5156 / 897.5265 / 854.5207
[b/c/a]$_{11}$	939.537 / 954.5479 / 911.5421	981.5476 / 996.5585 / 953.5527	981.5476 / 996.5585 / 953.5527	939.537 / 954.5479 / 911.5421
[b/c/a]$_{12}$	1010.5742 / 1025.585 / 982.5792	1052.5848 / 1067.5956 / 1024.5898	1052.5848 / 1067.5956 / 1024.5898	1010.5742 / 1025.585 / 982.5792
[b/c/a]$_{13}$	1180.6797 / 1195.6906 / 1152.6848	1180.6797 / 1195.6906 / 1152.6848	1180.6797 / 1195.6906 / 1152.6848	1180.6797 / 1195.6906 / 1152.6848
[y/z/x]$_{1}$	175.119 / 160.1081 / 203.1139	175.119 / 160.1081 / 203.1139	175.119 / 160.1081 / 203.1139	175.119 / 160.1081 / 203.1139
[y/z/x]$_{2}$	345.2245 / 330.2136 / 373.2194	303.2139 / 288.203 / 331.2088	303.2139 / 288.203 / 331.2088	345.2245 / 330.2136 / 373.2194
[y/z/x]$_{3}$	416.2616 / 401.2507 / 444.2565	374.251 / 359.2401 / 402.2459	374.251 / 359.2401 / 402.2459	416.2616 / 401.2507 / 444.2565
[y/z/x]$_{4}$	473.2831 / 458.2722 / 501.278	431.2725 / 416.2616 / 459.2674	431.2725 / 416.2616 / 459.2674	473.2831 / 458.2722 / 501.278
[y/z/x]$_{5}$	530.3045 / 515.2936 / 558.2995	488.2939 / 473.283 / 516.2889	488.2939 / 473.283 / 516.2889	530.3045 / 515.2936 / 558.2995
[y/z/x]$_{6}$	658.3995 / 643.3886 / 686.3944	658.3995 / 643.3886 / 686.3944	658.3995 / 643.3886 / 686.3944	658.3995 / 643.3886 / 686.3944
[y/z/x]$_{7}$	715.421 / 700.4101 / 743.4159	715.421 / 700.4101 / 743.4159	715.421 / 700.4101 / 743.4159	715.421 / 700.4101 / 743.4159
[y/z/x]$_{8}$	828.505 / 813.4941 / 856.4999	828.505 / 813.4941 / 856.4999	828.505 / 813.4941 / 856.4999	828.505 / 813.4941 / 856.4999
[y/z/x]$_{9}$	885.5265 / 870.5156 / 913.5214	885.5265 / 870.5156 / 913.5214	885.5265 / 870.5156 / 913.5214	885.5265 / 870.5156 / 913.5214
[y/z/x]$_{10}$	1013.6214 / 998.6106 / 1041.6164	1055.632 / 1040.6212 / 1083.627	1013.6214 / 998.6106 / 1041.6164	1055.632 / 1040.6212 / 1083.627
[y/z/x]$_{11}$	1070.6429 / 1055.632 / 1098.6378	1112.6535 / 1097.6426 / 1140.6484	1070.6429 / 1055.632 / 1098.6378	1112.6535 / 1097.6426 / 1140.6484
[y/z/x]$_{12}$	1127.6644 / 1112.6535 / 1155.6593	1169.675 / 1154.6641 / 1197.6699	1127.6644 / 1112.6535 / 1155.6593	1169.675 / 1154.6641 / 1197.6699
[y/z/x]$_{13}$	1297.7699 / 1282.759 / 1325.7648	1297.7699 / 1282.759 / 1325.7648	1297.7699 / 1282.759 / 1325.7648	1297.7699 / 1282.759 / 1325.7648
[b/c/a]$_{i(2-3)}$	228.1343 / 243.1452 / 200.1394	186.1237 / 201.1346 / 158.1288	228.1343 / 243.1452 / 200.1394	186.1237 / 201.1346 / 158.1288
[b/c/a]$_{i(2-4)}$	285.1558 / 300.1666 / 257.1608	243.1452 / 258.156 / 215.1502	285.1558 / 300.1666 / 257.1608	243.1452 / 258.156 / 215.1502
[b/c/a]$_{i(2-5)}$	413.2507 / 428.2616 / 385.2558	413.2507 / 428.2616 / 385.2558	413.2507 / 428.2616 / 385.2558	413.2507 / 428.2616 / 385.2558
[b/c/a]$_{i(2-6)}$	470.2722 / 485.2831 / 442.2773	470.2722 / 485.2831 / 442.2773	470.2722 / 485.2831 / 442.2773	470.2722 / 485.2831 / 442.2773
[b/c/a]$_{i(2-7)}$	583.3562 / 598.3671 / 555.3613	583.3562 / 598.3671 / 555.3613	583.3562 / 598.3671 / 555.3613	583.3562 / 598.3671 / 555.3613
[b/c/a]$_{i(2-8)}$	640.3777 / 655.3886 / 612.3828	640.3777 / 655.3886 / 612.3828	640.3777 / 655.3886 / 612.3828	640.3777 / 655.3886 / 612.3828
[b/c/a]$_{i(2-9)}$	768.4727 / 783.4836 / 740.4777	810.4833 / 825.4942 / 782.4883	810.4833 / 825.4942 / 782.4883	768.4727 / 783.4836 / 740.4777
[b/c/a]$_{i(2-10)}$	825.4941 / 840.505 / 797.4992	867.5047 / 882.5156 / 839.5098	867.5047 / 882.5156 / 839.5098	825.4941 / 840.505 / 797.4992
[b/c/a]$_{i(2-11)}$	882.5156 / 897.5265 / 854.5207	924.5262 / 939.5371 / 896.5313	924.5262 / 939.5371 / 896.5313	882.5156 / 897.5265 / 854.5207
[b/c/a]$_{i(2-12)}$	953.5527 / 968.5636 / 925.5578	995.5633 / 1010.5742 / 967.5684	995.5633 / 1010.5742 / 967.5684	953.5527 / 968.5636 / 925.5578
[b/c/a]$_{i(2-13)}$	1123.6583 / 1138.6691 / 1095.6633	1123.6582 / 1138.6691 / 1095.6633	1123.6582 / 1138.6691 / 1095.6633	1123.6583 / 1138.6691 / 1095.6633
[b/c/a]$_{i(3-4)}$	115.0502 / 130.0611 / 87.0553	115.0502 / 130.0611 / 87.0553	115.0502 / 130.0611 / 87.0553	115.0502 / 130.0611 / 87.0553
[b/c/a]$_{i(3-5)}$	243.1452 / 258.156 / 215.1502	285.1558 / 300.1666 / 257.1608	243.1452 / 258.156 / 215.1502	285.1558 / 300.1666 / 257.1608
[b/c/a]$_{i(3-6)}$	300.1666 / 315.1775 / 272.1717	342.1772 / 357.1881 / 314.1823	300.1666 / 315.1775 / 272.1717	342.1772 / 357.1881 / 314.1823
[b/c/a]$_{i(3-7)}$	413.2507 / 428.2616 / 385.2558	455.2613 / 470.2722 / 427.2664	413.2507 / 428.2616 / 385.2558	455.2613 / 470.2722 / 427.2664
[b/c/a]$_{i(3-8)}$	470.2721 / 485.283 / 442.2772	512.2827 / 527.2936 / 484.2878	470.2721 / 485.283 / 442.2772	512.2827 / 527.2936 / 484.2878
[b/c/a]$_{i(3-9)}$	598.3671 / 613.378 / 570.3722	682.3883 / 697.3992 / 654.3934	640.3777 / 655.3886 / 612.3828	640.3777 / 655.3886 / 612.3828
[b/c/a]$_{i(3-10)}$	655.3886 / 670.3994 / 627.3936	739.4098 / 754.4206 / 711.4148	697.3992 / 712.4101 / 669.4042	697.3992 / 712.41 / 669.4042
[b/c/a]$_{i(3-11)}$	712.41 / 727.4209 / 684.4151	796.4312 / 811.4421 / 768.4363	754.4206 / 769.4315 / 726.4257	754.4206 / 769.4315 / 726.4257
[b/c/a]$_{i(3-12)}$	783.4471 / 798.458 / 755.4522	867.4683 / 882.4792 / 839.4734	825.4577 / 840.4686 / 797.4628	825.4577 / 840.4686 / 797.4628
[b/c/a]$_{i(3-13)}$	953.5527 / 968.5636 / 925.5578	995.5633 / 1010.5742 / 967.5684	953.5527 / 968.5636 / 925.5578	995.5633 / 1010.5742 / 967.5684
[b/c/a]$_{i(4-5)}$	186.1237 / 201.1346 / 158.1288	228.1343 / 243.1452 / 200.1394	186.1237 / 201.1346 / 158.1288	228.1343 / 243.1452 / 200.1394
[b/c/a]$_{i(4-6)}$	243.1452 / 258.156 / 215.1502	285.1558 / 300.1666 / 257.1608	243.1452 / 258.156 / 215.1502	285.1558 / 300.1666 / 257.1608
[b/c/a]$_{i(4-7)}$	356.2292 / 371.2401 / 328.2343	398.2398 / 413.2507 / 370.2449	356.2292 / 371.2401 / 328.2343	398.2398 / 413.2507 / 370.2449
[b/c/a]$_{i(4-8)}$	413.2507 / 428.2616 / 385.2558	455.2613 / 470.2722 / 427.2664	413.2507 / 428.2616 / 385.2558	455.2613 / 470.2722 / 427.2664
[b/c/a]$_{i(4-9)}$	541.3456 / 556.3565 / 513.3507	625.3668 / 640.3777 / 597.3719	583.3562 / 598.3671 / 555.3613	583.3562 / 598.3671 / 555.3613
[b/c/a]$_{i(4-10)}$	598.3671 / 613.378 / 570.3722	682.3883 / 697.3992 / 654.3934	640.3777 / 655.3886 / 612.3828	640.3777 / 655.3886 / 612.3828
[b/c/a]$_{i(4-11)}$	655.3886 / 670.3995 / 627.3936	739.4098 / 754.4206 / 711.4148	697.3992 / 712.4101 / 669.4042	697.3992 / 712.4101 / 669.4042
[b/c/a]$_{i(4-12)}$	726.4257 / 741.4366 / 698.4308	810.4469 / 825.4578 / 782.452	768.4363 / 783.4472 / 740.4414	768.4363 / 783.4472 / 740.4414
[b/c/a]$_{i(4-13)}$	896.5312 / 911.5421 / 868.5363	938.5418 / 953.5527 / 910.5469	896.5312 / 911.5421 / 868.5363	938.5418 / 953.5527 / 910.5469
[b/c/a]$_{i(5-6)}$	186.1237 / 201.1346 / 158.1288	228.1343 / 243.1452 / 200.1394	186.1237 / 201.1346 / 158.1288	228.1343 / 243.1452 / 200.1394
[b/c/a]$_{i(5-7)}$	299.2078 / 314.2186 / 271.2128	341.2184 / 356.2292 / 313.2234	299.2078 / 314.2186 / 271.2128	341.2184 / 356.2292 / 313.2234
[b/c/a]$_{i(5-8)}$	356.2292 / 371.2401 / 328.2343	398.2398 / 413.2507 / 370.2449	356.2292 / 371.2401 / 328.2343	398.2398 / 413.2507 / 370.2449
[b/c/a]$_{i(5-9)}$	484.3242 / 499.3351 / 456.3293	568.3454 / 583.3563 / 540.3505	526.3348 / 541.3457 / 498.3399	526.3348 / 541.3457 / 498.3399

$[b/c/a]_{i(5-10)}$	541.3456 / 556.3565 / 513.3507	625.3668 / 640.3777 / 597.3719	583.3562 / 598.3671 / 555.3613	583.3562 / 598.3671 / 555.3613
$[b/c/a]_{i(5-11)}$	598.3671 / 613.378 / 570.3722	682.3883 / 697.3992 / 654.3934	640.3777 / 655.3886 / 612.3828	640.3777 / 655.3886 / 612.3828
$[b/c/a]_{i(5-12)}$	669.4042 / 684.4151 / 641.4093	753.4254 / 768.4363 / 725.4305	711.4148 / 726.4257 / 683.4199	711.4148 / 726.4257 / 683.4199
$[b/c/a]_{i(5-13)}$	839.5098 / 854.5207 / 811.5148	881.5204 / 896.5313 / 853.5254	839.5098 / 854.5207 / 811.5148	881.5204 / 896.5313 / 853.5254
$[b/c/a]_{i(6-7)}$	171.1128 / 186.1237 / 143.1179	171.1128 / 186.1237 / 143.1179	171.1128 / 186.1237 / 143.1179	171.1128 / 186.1237 / 143.1179
$[b/c/a]_{i(6-8)}$	228.1343 / 243.1452 / 200.1393	228.1343 / 243.1452 / 200.1393	228.1343 / 243.1452 / 200.1393	228.1343 / 243.1452 / 200.1393
$[b/c/a]_{i(6-9)}$	356.2292 / 371.2401 / 328.2343	398.2398 / 413.2507 / 370.2449	398.2398 / 413.2507 / 370.2449	356.2292 / 371.2401 / 328.2343
$[b/c/a]_{i(6-10)}$	413.2507 / 428.2616 / 385.2558	455.2613 / 470.2722 / 427.2664	455.2613 / 470.2722 / 427.2664	413.2507 / 428.2616 / 385.2558
$[b/c/a]_{i(6-11)}$	470.2721 / 485.283 / 442.2772	512.2827 / 527.2936 / 484.2878	512.2827 / 527.2936 / 484.2878	470.2721 / 485.283 / 442.2772
$[b/c/a]_{i(6-12)}$	541.3092 / 556.3201 / 513.3143	583.3198 / 598.3307 / 555.3249	583.3198 / 598.3307 / 555.3249	541.3092 / 556.3201 / 513.3143
$[b/c/a]_{i(6-13)}$	711.4148 / 726.4257 / 683.4199	711.4148 / 726.4257 / 683.4199	711.4148 / 726.4257 / 683.4199	711.4148 / 726.4257 / 683.4199
$[b/c/a]_{i(7-8)}$	171.1128 / 186.1237 / 143.1179	171.1128 / 186.1237 / 143.1179	171.1128 / 186.1237 / 143.1179	171.1128 / 186.1237 / 143.1179
$[b/c/a]_{i(7-9)}$	299.2078 / 314.2186 / 271.2128	341.2184 / 356.2292 / 313.2234	341.2184 / 356.2292 / 313.2234	299.2078 / 314.2186 / 271.2128
$[b/c/a]_{i(7-10)}$	356.2292 / 371.2401 / 328.2343	398.2398 / 413.2507 / 370.2449	398.2398 / 413.2507 / 370.2449	356.2292 / 371.2401 / 328.2343
$[b/c/a]_{i(7-11)}$	413.2507 / 428.2616 / 385.2558	455.2613 / 470.2722 / 427.2664	455.2613 / 470.2722 / 427.2664	413.2507 / 428.2616 / 385.2558
$[b/c/a]_{i(7-12)}$	484.2878 / 499.2987 / 456.2929	526.2984 / 541.3093 / 498.3035	526.2984 / 541.3093 / 498.3035	484.2878 / 499.2987 / 456.2929
$[b/c/a]_{i(7-13)}$	654.3933 / 669.4042 / 626.3984	654.3933 / 669.4042 / 626.3984	654.3933 / 669.4042 / 626.3984	654.3933 / 669.4042 / 626.3984
$[b/c/a]_{i(8-9)}$	186.1237 / 201.1346 / 158.1288	228.1343 / 243.1452 / 200.1394	228.1343 / 243.1452 / 200.1394	186.1237 / 201.1346 / 158.1288
$[b/c/a]_{i(8-10)}$	243.1452 / 258.156 / 215.1502	285.1558 / 300.1666 / 257.1608	285.1558 / 300.1666 / 257.1608	243.1452 / 258.156 / 215.1502
$[b/c/a]_{i(8-11)}$	300.1666 / 315.1775 / 272.1717	342.1772 / 357.1881 / 314.1823	342.1772 / 357.1881 / 314.1823	300.1666 / 315.1775 / 272.1717
$[b/c/a]_{i(8-12)}$	371.2037 / 386.2146 / 343.2088	413.2143 / 428.2252 / 385.2194	413.2143 / 428.2252 / 385.2194	371.2037 / 386.2146 / 343.2088
$[b/c/a]_{i(8-13)}$	541.3093 / 556.3202 / 513.3144	541.3093 / 556.3202 / 513.3144	541.3093 / 556.3202 / 513.3144	541.3093 / 556.3202 / 513.3144
$[b/c/a]_{i(9-10)}$	186.1237 / 201.1346 / 158.1288	228.1343 / 243.1452 / 200.1394	228.1343 / 243.1452 / 200.1394	186.1237 / 201.1346 / 158.1288
$[b/c/a]_{i(9-11)}$	243.1452 / 258.156 / 215.1502	285.1558 / 300.1666 / 257.1608	285.1558 / 300.1666 / 257.1608	243.1452 / 258.156 / 215.1502
$[b/c/a]_{i(9-12)}$	314.1823 / 329.1932 / 286.1874	356.1929 / 371.2038 / 328.1979	356.1929 / 371.2038 / 328.1979	314.1823 / 329.1932 / 286.1874
$[b/c/a]_{i(9-13)}$	484.2878 / 499.2987 / 456.2929	484.2878 / 499.2987 / 456.2929	484.2878 / 499.2987 / 456.2929	484.2878 / 499.2987 / 456.2929
$[b/c/a]_{i(10-11)}$	115.0502 / 130.0611 / 87.0553	115.0502 / 130.0611 / 87.0553	115.0502 / 130.0611 / 87.0553	115.0502 / 130.0611 / 87.0553
$[b/c/a]_{i(10-12)}$	186.0873 / 201.0982 / 158.0924	186.0873 / 201.0982 / 158.0924	186.0873 / 201.0982 / 158.0924	186.0873 / 201.0982 / 158.0924
$[b/c/a]_{i(10-13)}$	356.1929 / 371.2038 / 328.1979	314.1823 / 329.1932 / 286.1874	314.1823 / 329.1932 / 286.1874	356.1929 / 371.2038 / 328.1979
$[b/c/a]_{i(11-12)}$	129.0659 / 144.0767 / 101.0709	129.0659 / 144.0767 / 101.0709	129.0659 / 144.0767 / 101.0709	129.0659 / 144.0767 / 101.0709
$[b/c/a]_{i(11-13)}$	299.1714 / 314.1823 / 271.1765	257.1608 / 272.1717 / 229.1659	257.1608 / 272.1717 / 229.1659	299.1714 / 314.1823 / 271.1765
$[b/c/a]_{i(12-13)}$	242.1499 / 257.1608 / 214.155	200.1394 / 215.1502 / 172.1444	200.1394 / 215.1502 / 172.1444	242.1499 / 257.1608 / 214.155

2. 2D-PC-MS experimental data for mixtures of co-fragmented $[M+3H]^{3+}$ ions of the diacetylated histone H4 fragment 4–17 isomers.

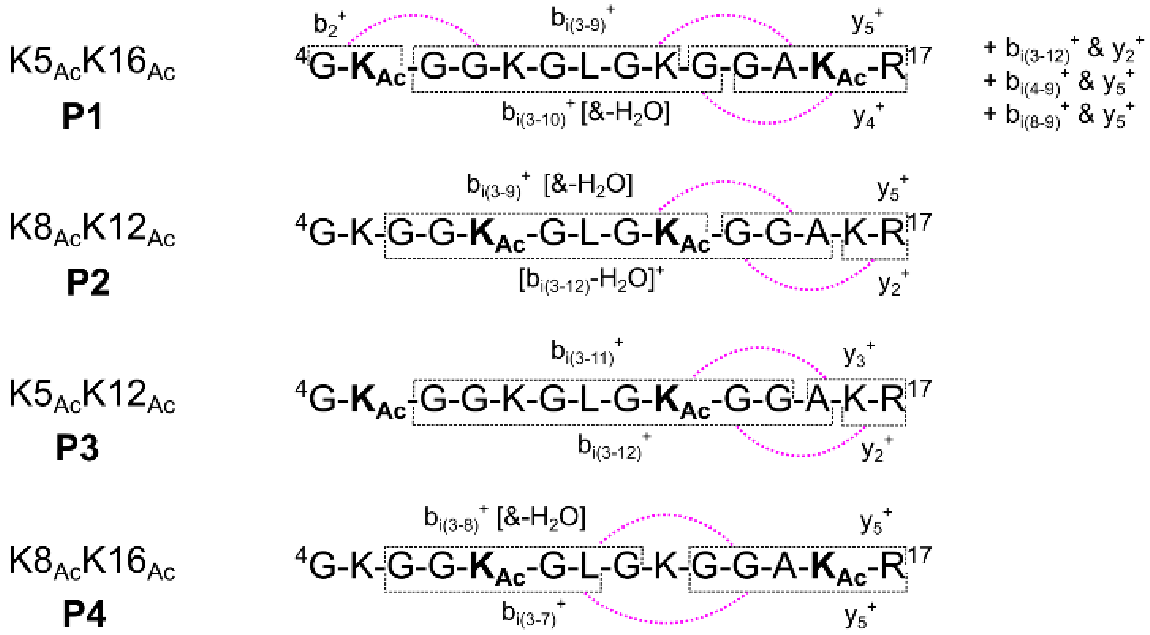

Figure S1. The 2D-PC-MS marker ion correlations for co-fragmenting $[M+3H]^{3+}$ ions of the combinatorial isomers **P1**-**P4** under LIT-CID. The marker fragment-fragment ion correlations enable the unambiguous detection of each individual isomer within the mixture. The isomer-specific pairs of correlating fragments are designated by horizonal brackets, and their correlations are shown by magenta arcs. Each of the measured marker ion correlations are of internal-terminal type, confirming the previously predicted supreme sequence specificity of 2D-PC-MS correlations which involve internal fragment ions [13].

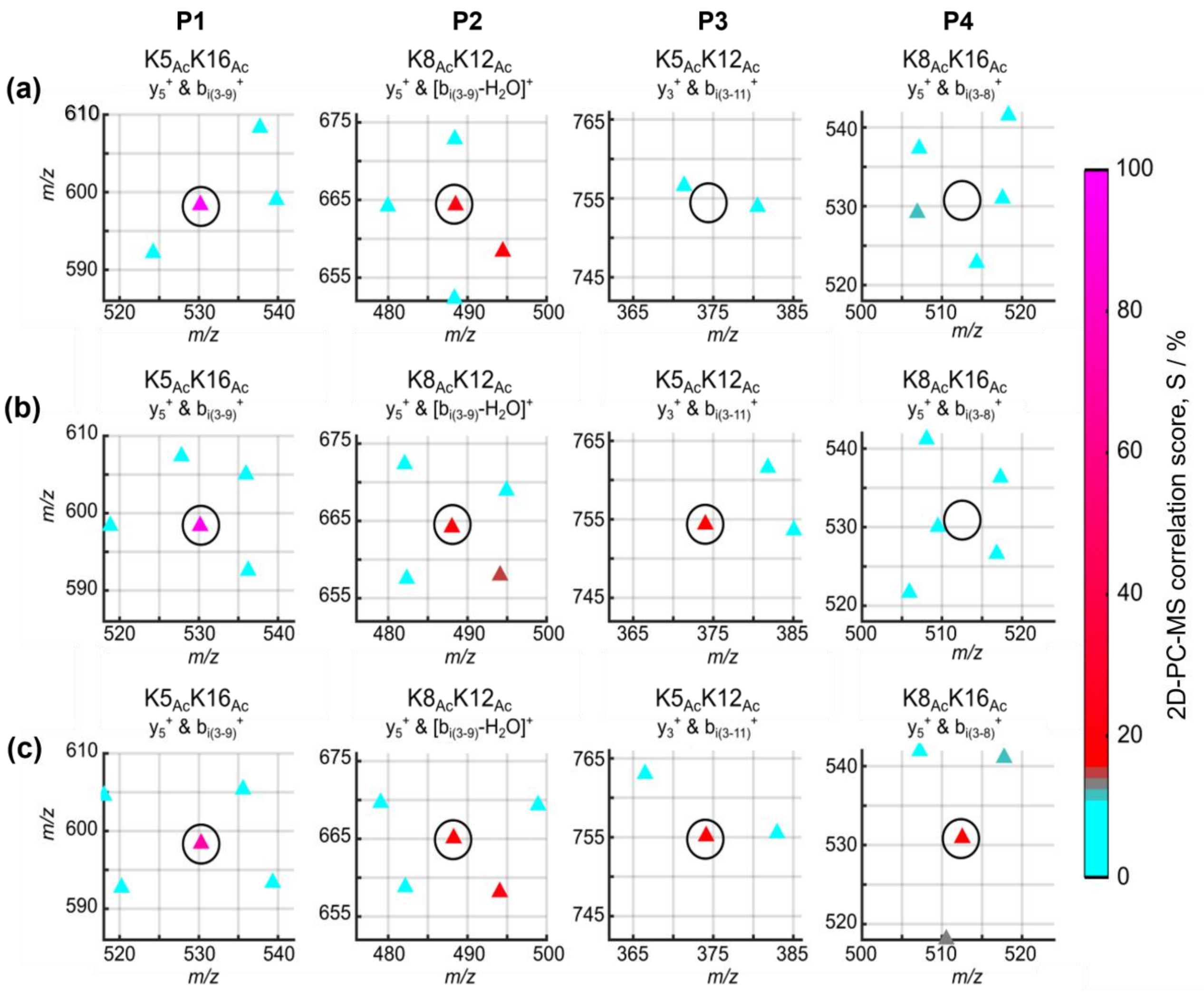

Figure S2. Identification of components of the mixtures of (a) two, (b) three and (c) four combinatorial isomers **P1**-**P4** using 2D-PC-MS. For each isomer, only one of the marker ion correlations given in Figure S1 is displayed using the correlation scores (as shown in Figure 2b of the main text). The high-ranking correlations (see the color scale) reveal unambiguously the presence of a corresponding isomer in a sample.

3. Quantitative analysis of the combinatorial isomer mixture using 2D-PC-MS

In addition to providing a method to qualitatively discriminate between different combinatorial isomers, the self-correcting partial covariance between two fragment ion signals provides a quantitative measurement of the abundance of the peptide under analysis. This follows from the theory of partial covariance mapping using the total ion count as a single correction parameter, as presented in [13]. According to the theory, the measured value of any marker ion correlation provides a quantitative measure of the concentration of the corresponding parent ion. In principle, this requires monitoring of only one marker ion correlation for the quantification of its specific isomer. Fig. S3 shows the experimental verification of this theoretical prediction. It displays the measured value of partial covariance between fragments $y_{5,Ac}^{+}$ & $b_{i(3\text{-}9)}^{+}$, which is a marker ion correlation of the isomer $K_{5,Ac}K_{16,Ac}$. The relative concentration of this isomer ('Quantity added') has been increased in a mixture of four combinatorial isomers and the measured value ('Quantity measured') of partial covariance rises linearly with the increased concentration. The relative concentration of a particular isomer is obtained by dividing the volume of its marker ion correlation peak by the volume of any reference partial covariance peak, stemming from another isomer, which cannot have been produced by the parent ion in question. The error bars show the dependence of measured value on the reference peak chosen. This demonstration paves the way for the quantification of the combinatorial isomers in their mixtures by 2D-PC-MS.

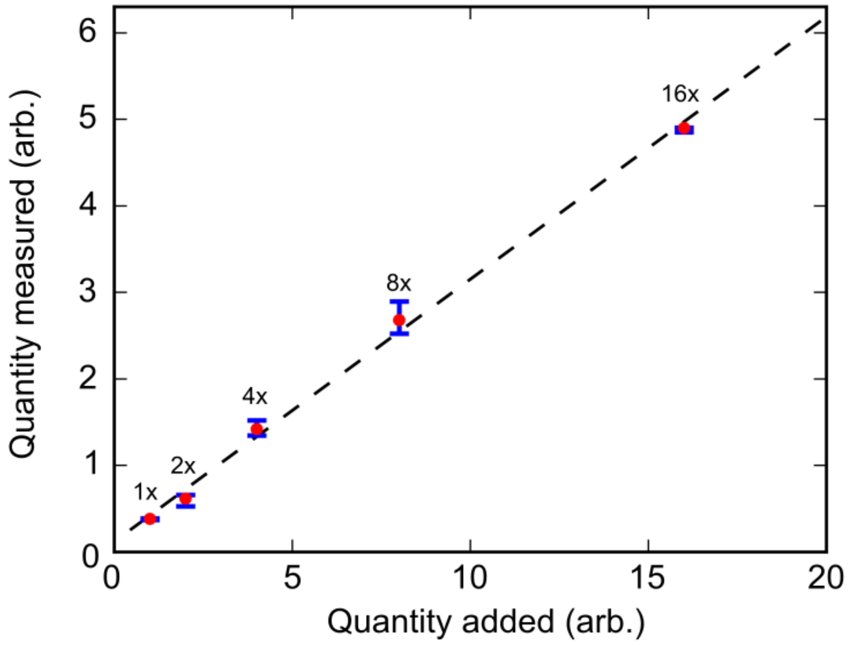

Figure S3. Linear mapping of the measured value of partial covariance between fragments $y_{5,Ac}^{+}$ & $b_{i(3\text{-}9)}^{+}$ with concentration of isomer $K_{5,Ac}K_{16,Ac}$, in a mixture of all four combinatorial isomers.

4. *In silico* identification of the MS/MS marker fragments and 2D-PC-MS marker fragment-fragment correlations in human histone tail peptides

Histone proteins can be digested by trypsin following propionic anhydride derivatization that prevents cleavage C-terminal to lysine and leads to cleavages C-terminal to arginine exclusively or by Glu-C leading to cleavages on C-terminus of either aspartic or aspartic and glutamic acids residues, see https://www.sigmaaldrich.com/GB/en/product/sigma/p6181. Here we consider all the possible multiply acetylated isomers of the peptides that could be produced by tryptic or Glu-C digestion of human histones H1, H2A, H2B, H3 and H4. Table S3 below summarises the resulting peptide sequences derived from the Swissprot database, https://www.uniprot.org/.

Table S3. Peptide sequences resulting from tryptic and Glu-C digestion of human histones H1, H2A, H2B, H3 and H4. Lysine (K) residues – the possible acetylation sites – are highlighted in bold. Note that upon trypsin digestion, the H3 histones do not produce long enough peptides that could exhibit combinatorial acetylation.

Histone	Tryptic peptides	Glu-C peptides
H1	A**K**AS**KK**STDHP**K**YSDMIVAAIQ<br>AE**K**NR	NSTSAPAA**K**P**K**RA**K**AS**KK**STD<br><br>NSTSAPAA**K**P**K**RA**K**AS**KK**ST**D**HP**K**YSDMIVAAIQAE
H2B	PEPA**K**SAPAP**KK**GS**KK**AVTKAQ<br>**KK**DG**KK**R	PA**K**SAPAP**KK**GS**KK**AVTKAQ**KK**DG**KK**R**K**RSR**K**E
H2A	AGG**K**AG**K**DSG**K**A**K**A**K**AVSR	AGG**K**AG**K**DSG**K**A**K**A**K**AVSRSQRAGLQFPVGRIHRHL**K**TRTTSHG<br>RVGATAAVYSAAILE
H3	-	ART**K**QTAR**K**STGG**K**APR**K**QLAT**K**AAR**K**SAPATGGV**KK**PHRYRPG<br>TVALRE
H4	G**K**GG**K**GLG**K**GGA**K**R	SGRG**K**GG**K**GLG**K**GGA**K**RHR**K**VLRD<br><br>SGRG**K**GG**K**GLG**K**GGA**K**RHR**K**VLRDNIQGIT**K**PAIRRLARRGGVK<br>RISGLIYE

In our simulations, we have considered all the acetylation states of each of the sequences in Table S3, apart from non-acetylated and fully acetylated cases that naturally do not lead to positional isomerism. For each acetylation state of each sequence, we assumed co-fragmentation of all the possible positional isomers. We have evaluated the mean fragment/fragment pair ambiguity rate, FAR, for individual fragments (1D MS/MS) as well as for fragment-fragment correlations (2D-PC-MS) for each of the positional isomers, defining it as in Appendix D of Ref. [13] on the "database" of the positional isomers with the same number of acetylated sites. Specifically, for every theoretical fragment ion (1D MS/MS) or fragment ion correlation (2D-PC-MS) x, the fragment/fragment pair ambiguity rate (FAR) value was calculated according to the following expression:

$$R_{FAR}(x) = \frac{\sum_P N_m(P,T,x)}{N_P} \qquad \text{(S1)}$$

where the summation is over all the N_P combinatorial isomers of the parent ion of the fragment ion (or fragment-fragment correlation) x. $N_m(P,T,x)$ is the number of *m/z* matches, within the fragment *m/z* tolerance, T, here assumed to be 1e-8 Da (corresponding to exactly matching masses, $R_{FAR}(x)$ results were found to be almost identical to the ones at m/z tolerance of 0.02 Da), between x and all the possible fragment ions (or fragment ion correlations) of the fragmenting isomer P.

Below is a series of results of such calculations for the multiply acetylated Glu-C peptide of H2A. Marker fragments (1D MS/MS, blue stars in Figs. S4-S7) were identified as either terminal or internal fragments that are unique in their *m/z* ratio to a single positional isomer. Marker fragment correlations (2D-PC-MS, red stars in Figs. S4-S7) were identified as pairs of correlating terminal or internal ions that are unique in their (m_1/z_1, m_2/z_2) ratios to a single positional isomer.

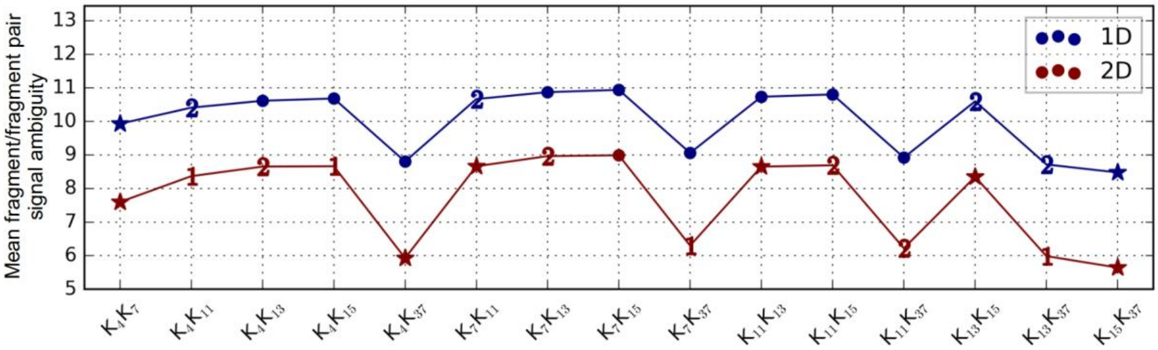

Figure S4. Mean ambiguity rate of the individual fragments (1D MS/MS, blue) and fragment-fragment correlations (2D-PC-MS, red) of the doubly acetylated isomers of Glu-C peptide of H2A histone, see Table S3 for the peptide sequence. Cases where there exists a marker fragment or a marker fragment-fragment correlation are highlighted by stars; cases where the fragments or fragment-fragment correlations allow one to identify a pair of isomers are marked by “1”; cases where the fragments or fragment-fragment correlations allow one to identify a triplet of isomers are marked by “2”. 2D-PC-MS uniformly yields higher specificity across all isomers with all but a single isomer out of 15 being identified either uniquely or as one of a pair or as one of a triplet of positional isomers.

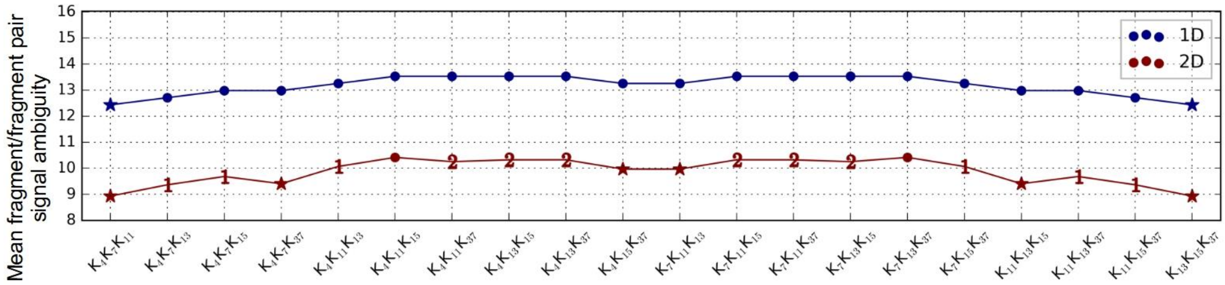

Figure S5. Same as Fig. S4 for triply acetylated isomers of Glu-C peptide of H2A histone, see Table S3 for the peptide sequence.

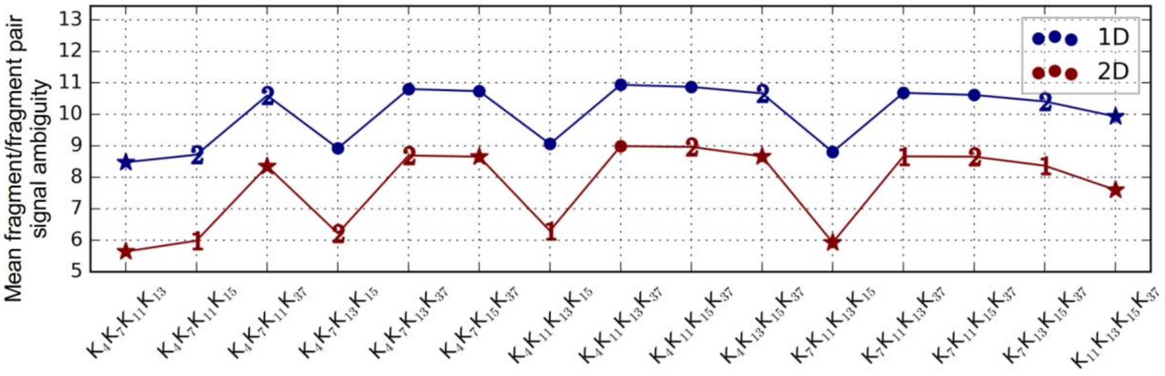

Figure S6. Same as Fig. S4 for quadruply acetylated isomers of Glu-C peptide of H2A histone, see Table S3 for the peptide sequence.

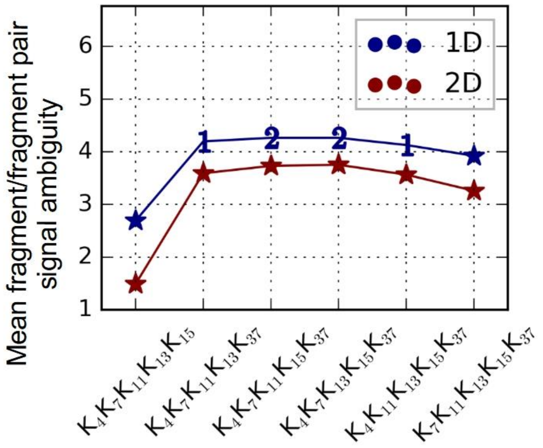

Figure S7. Same as Fig. S4 for quintuply acetylated isomers of Glu-C peptide of H2A histone, see Table S3 for the peptide sequence.

Having considered all the combinatorial positional isomers of the peptides in Table S3, we have compiled the numbers of the uniquely identified isomers for the tryptic and Glu-C peptides of each of the histones into a histogram presented in Fig. 3 of the main text. Here we show the analogous histograms for the numbers of isomers that could be identified as one of a pair (Fig. S8) or one of a triplet (Fig. S9). Figures S8 and S9 show that on top of the three- to five-fold increase in the unique isomer identifications (see Fig. 3 of the main text), 2D-PC-MS overwhelmingly outperforms 1D MS/MS in the identifications of pairs and triplets of isomers.

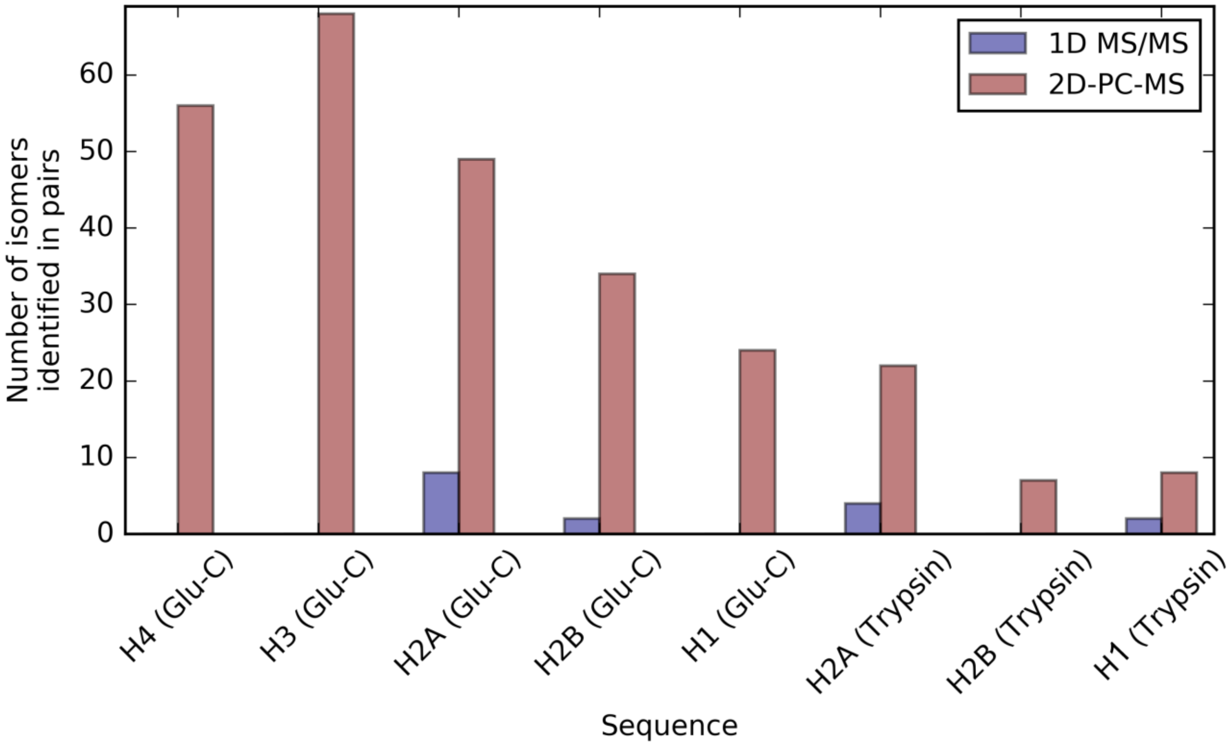

Figure S8. Total number of the combinatorial isomers of acetylated Glu-C and tryptic peptides of human histones that can be identified in their mixtures as one of a pair of isomers by 1D MS/MS (blue) and by 2D-PC-MS (red).

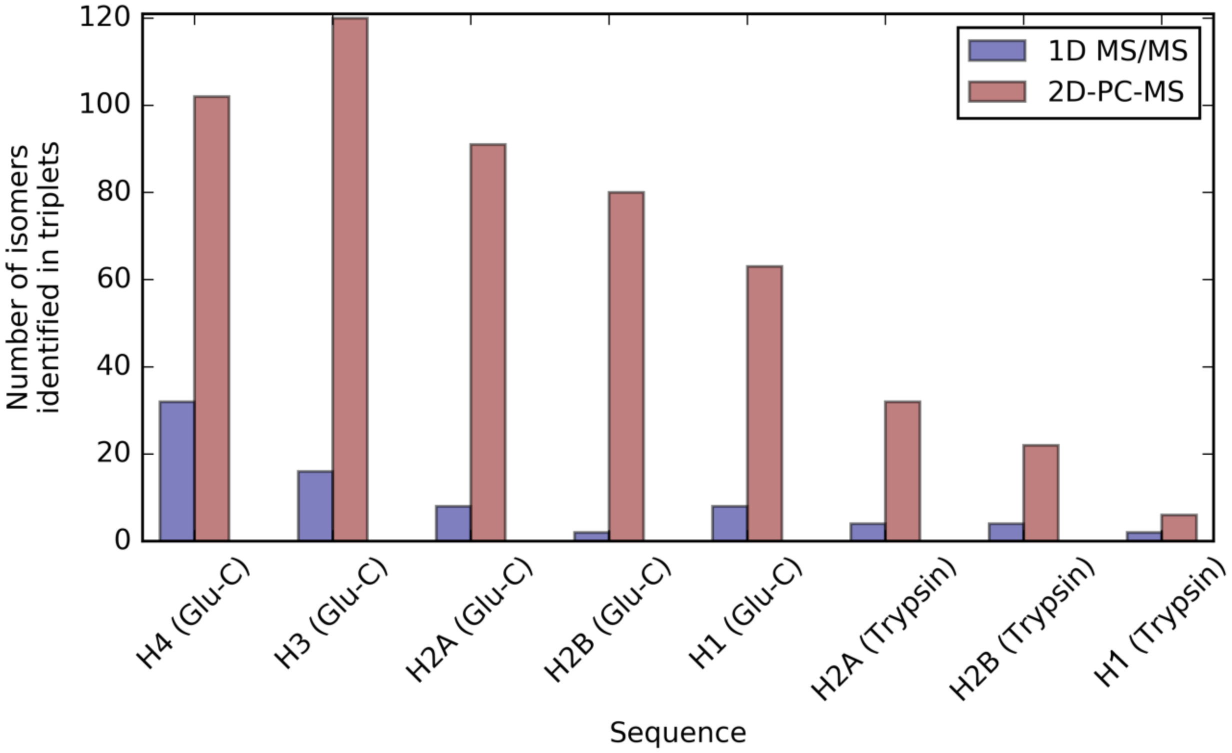

Figure S9. Total number of the combinatorial isomers of acetylated Glu-C and tryptic peptides of human histones that can be identified in their mixtures as one of a triplet of isomers by 1D MS/MS (blue) and by 2D-PC-MS (red).
